# Supplementary material for: Probing the electronic structure and photophysics of thiophene–diketopyrrolopyrrole derivatives in solution
Source: Phys Chem Chem Phys. 2022 Aug 15;24(34):20138–51. doi: 10.1039/d2cp03238d (PMC9429679; doi:10.1039/d2cp03238d)
Supplement: CP-024-D2CP03238D-s001 [file CP-024-D2CP03238D-s001.pdf]

Electronic Supplementary Material (ESI) for Physical Chemistry Chemical Physics.
This journal is © the Owner Societies 2022

Supporting information for:
Probing the electronic structure and photophysics of thiophene–diketopyrrolopyrrole derivatives in solution

Daniel W. Polak,[1] Mariana T. do Casal,[2] Josene M. Toldo,[2] Xiantao Hu,[3] Giordano Amoruso,[1] Olivia Pomeranc,[1] Martin Heeney,[3] Mario Barbatti,[2,4] Michael N. R. Ashfold,[1] and Thomas A. A. Oliver[1]*

[1] School of Chemistry, Cantock's Close, University of Bristol, Bristol, BS8 1TS, UK

[2] Aix-Marseille Université, CNRS, ICR, Marseille, France

[3] Department of Chemistry and Centre for Processable Electronics, Imperial College London, White City Campus, London, W12 0BZ, UK

[4] Institut Universitaire de France, 75231, Paris, France

Author for correspondence: tom.oliver@bristol.ac.uk

1. Laser Pump Pulse Characterisation

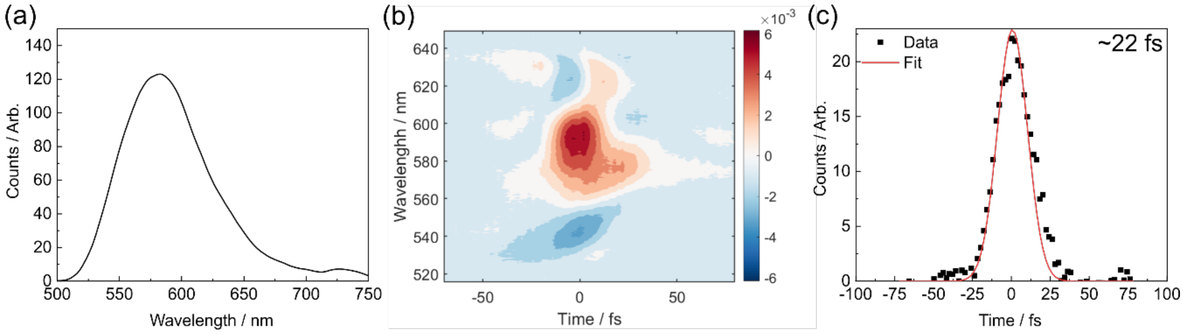

Fig. S1 (a) Pump laser spectrum used in TA measurements, (b) Polarised-gating frequency resolved optical gating (PG-FROG) characterisation of pump pulses, (c) autocorrelation trace extracted from PG-FROG spectrum (square points) overlaid with Gaussian fit to data (red solid line).

2. Transient Absorption Spectroscopy

(a) Wavepacket analysis of Toluene

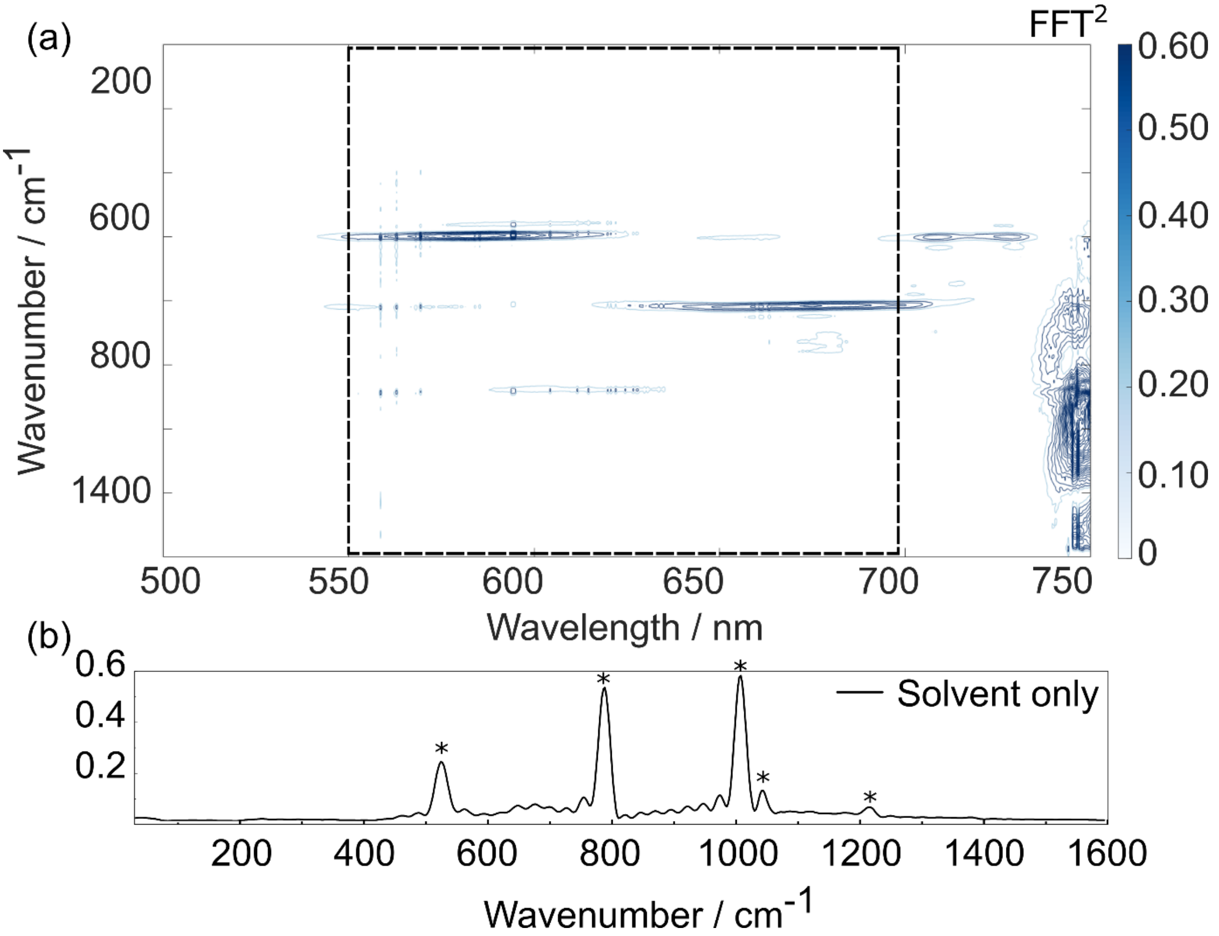

Fig. S2 Toluene non-resonant impulse stimulated Raman scattering (ISRS) wavepacket dynamics. (a) False colour map of vibrational wavepacket wavenumbers as a function of probe wavelength. (b) Vibrational wavenumbers associated with toluene averaged over the 550–700 nm window. A list of the observed wavenumbers associated with toluene signals is given in Table S1.

Table S1 Wavenumbers associated with peaks in the averaged toluene spectrum shown in Fig. S2 compared to previously experimentally determined Raman wavenumbers of toluene (from refs [1,2]).

Observed Wavenumber / cm^{-1}	Literature value / cm^{-1}
523	522
784	787
1000	1004
1032	1031
1208	1211

(b) DPPDTT in Toluene

The main manuscript details transient measurements of DPPDTT acquired in chloroform solution. The equivalent data acquired in toluene are displayed in Fig. S3(a). Due to the low solubility of the polymer and some precipitation in toluene, significant scattering is observed in the associated TA data, resulting in far lower signal-to-noise ratios compared to data obtained in chloroform (Fig. 7). To alleviate some of these issues, the frequency and time domains were smoothed by adjacent averaging and the use of a Savitzky-Golay filter, respectively. A comparison of the kinetics for DPPDTT in the two solvents for three probe wavelengths are shown in Fig. S3(b-d), and show clear similarities.

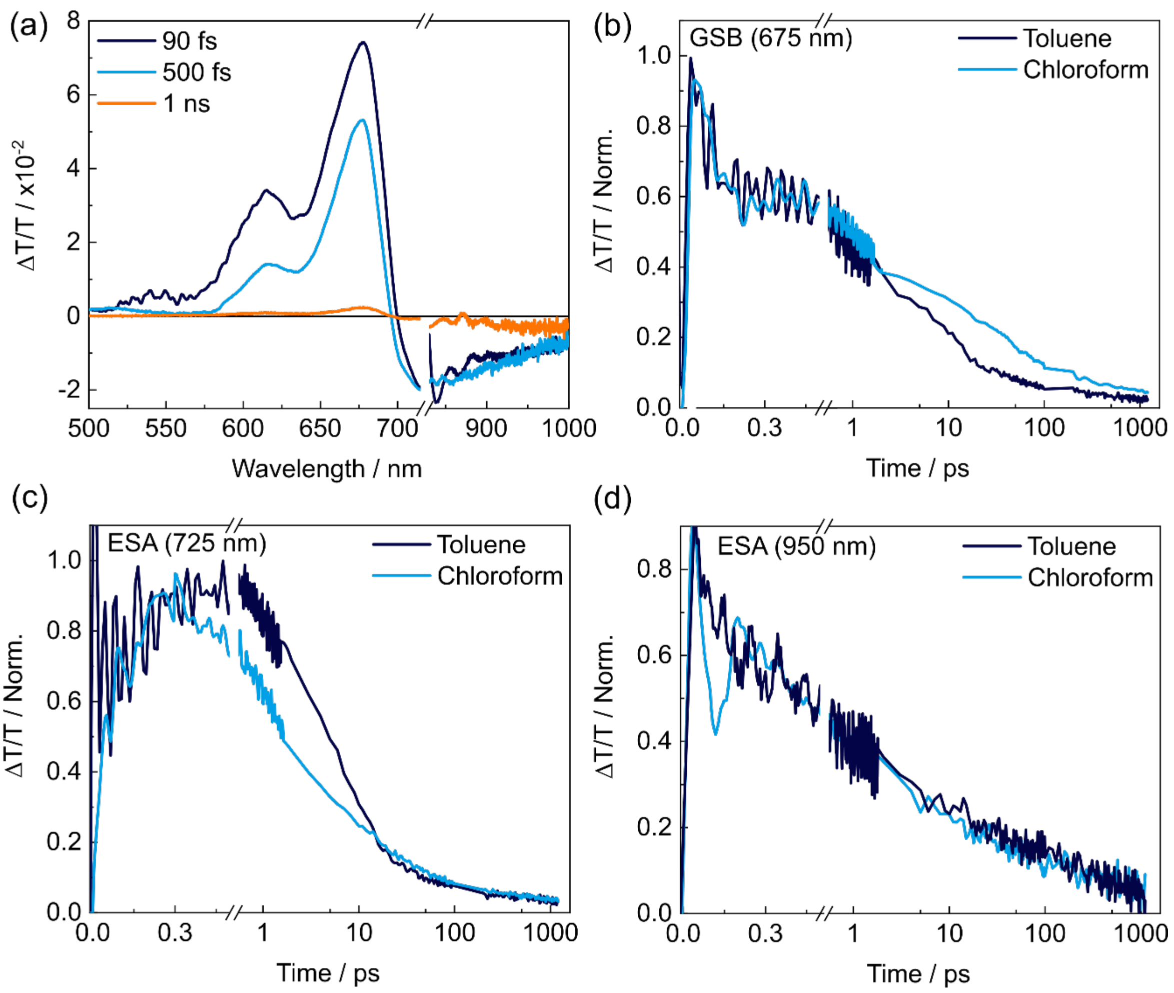

Fig. S3 (a) DPPDTT transient absorption spectra in toluene solution and (b-d) comparison of kinetics in toluene and chloroform for three probe wavelengths. Toluene kinetics have been smoothed via application of a Savitzky-Golay filter.

(c) DPPDTT Rate Model

A rate model for both chloroform and toluene measurements was constructed to dissect the complex polymer dynamics: the photoexcited kinetics of DPPDTT were described using a generic rate model (eq. S1), where $y(n)$ and $y(m)$ are the population of the nth and m^{th} states. k_m is the decay rate constant for $y(m)$, which also describes the rate of $y(n)$ formation. The population of $y(n)$ subsequently decays with rate constant k_n. Combined, the time dependent population of $y(n)$ can be described:

$$\frac{dy(n)}{dt} = k_m y(m) - k_n y(n)\,. \qquad \text{(S1)}$$

The model includes three electronic states: The ground state (S_0), a bright state which is optically prepared (S_{bright}) and a dark intermediate state (S_{dark}). S_{bright} is generated with a generation function, G, that describes the IRF and also serves to depopulate S_0. S_{bright} can subsequently convert into S_{dark}, and both states subsequently decay in parallel back to S_0. The model assumes that there are several independent populations of each state generated which do not interconvert on the picosecond time scale as justified in the text below. The constructed equations are then solved numerically using a Matlab script.

There are two possible origins for the range of lifetimes observed in the polymer, arising from either large-scale conformational inhomogeneity, such as planar *vs.* twisted polymer chains, or differences in conjugation lengths on different chain segments. As discussed in the main text, because the absorption maximum of the polymer matches that of the dimer, the delocalisation is considered to be spread over ~2 repeat units (Fig. 1(d))- a conclusion supported by a recent computational study of DPPDTT.[3] Further, if a range of effective conjugation lengths were present, significant broadening of the absorption spectrum would be expected and the evident vibronic progression would not be well resolved. Together this suggests the observed lifetimes represent different sub-ensemble populations corresponding to different polymer geometries (all affording a delocalisation length scale of ~2 repeat units) each with a unique environment. These conformations are static on the timescale of the experiment but have differing photophysical properties leading to the range of reported lifetimes.

The kinetic fits to chloroform data are displayed/discussed in the main text (Fig. 7). A similar model was constructed for DPPDTT in toluene, and the results from this analysis are displayed in Fig. S4. Notably some of the time constants are altered, which is proposed to be due to slight changes in the relative energies of the electronic states, however no additional kinetic components are required to describe the data. Given the excellent match between experimental data and modelled kinetics, it is evident that a three-state model robustly describes the excited state dynamics of DPPDTT.

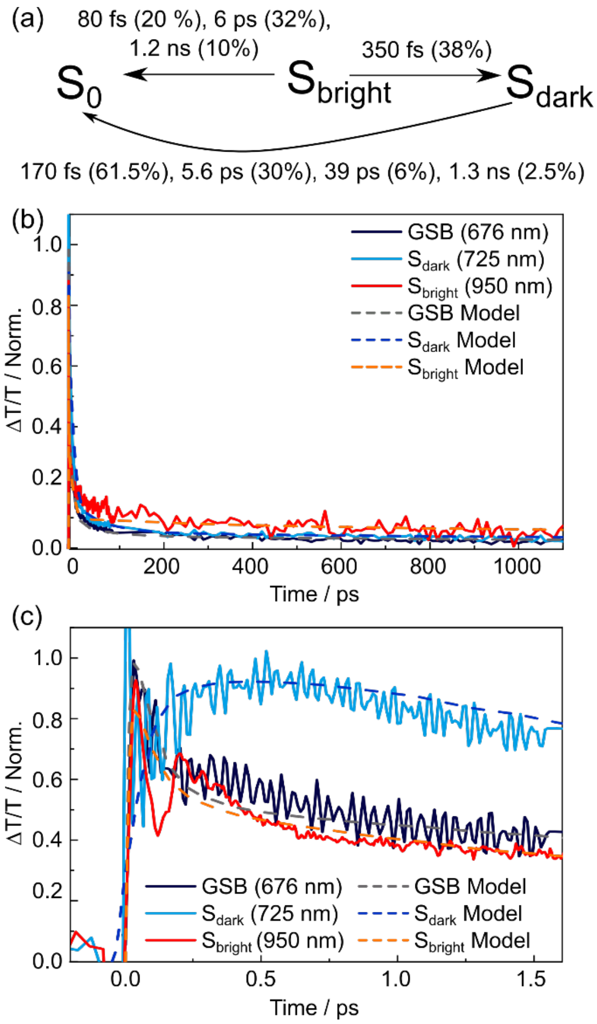

Fig. S4 (a) Summary of the rate model used to describe DPPDTT kinetics in toluene solution. The associated amplitudes with each lifetime component (as percentages) are given in brackets, the amplitudes have been normalised to match the GSB kinetics. Normalised kinetics for the (b) nanosecond and (c) picosecond dynamic regimes, with overlaid fits (dashed lines) returned from rate model.

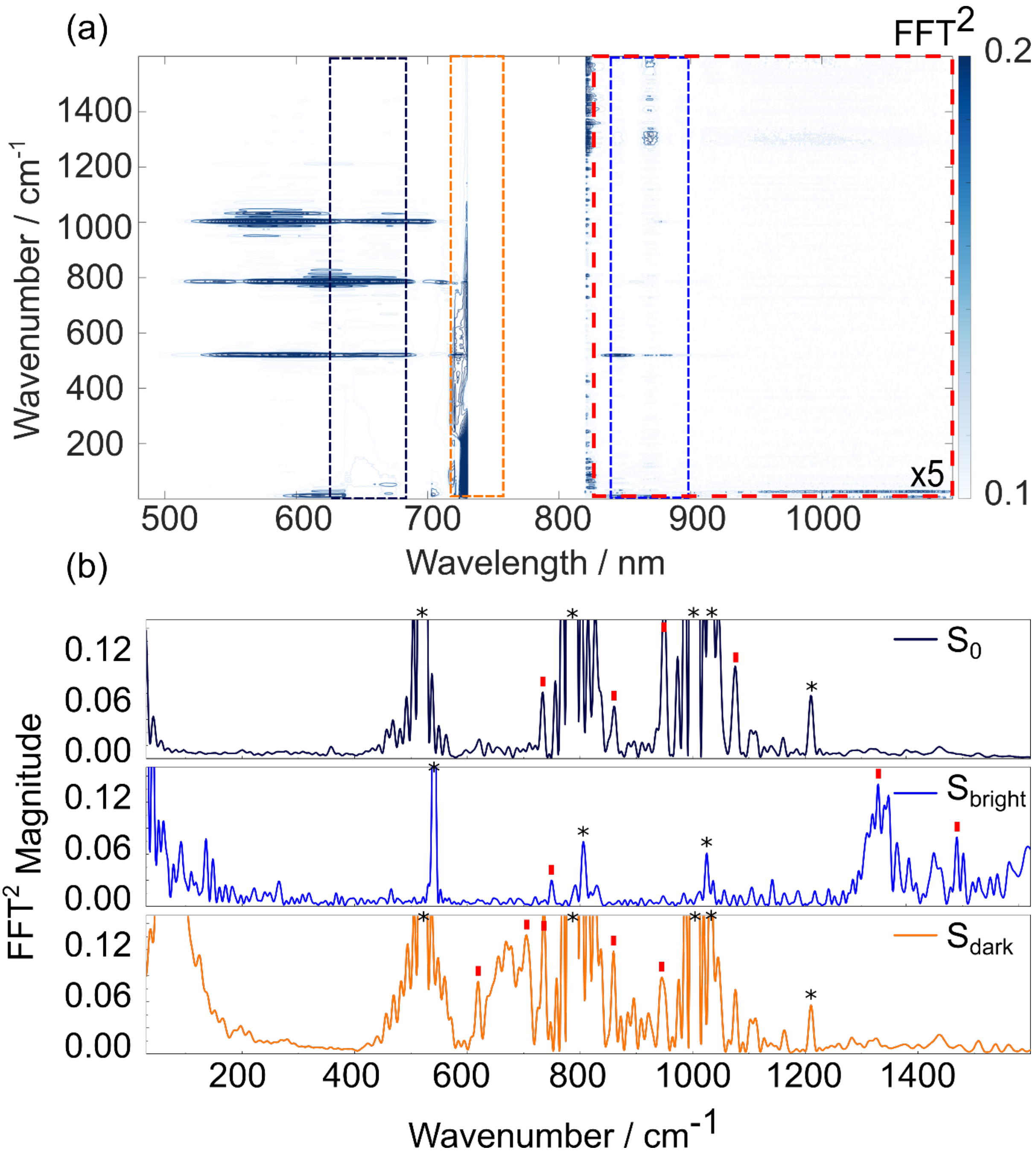

Fig. S5 Wavepacket dynamics of DPPDTT in toluene solution. (a) False colour map of vibrational wavenumber *vs.* probe wavelength extracted from transient absorption data, (b) vibrational wavenumbers associated with the GSB (625–675 nm), and near-IR ESAs (710–730 nm and 850–900 nm). Asterisks indicate solvent vibrations; red tick labels indicate assigned polymer modes discussed in section 8 of the ESI.

3. Kinetic Fitting Parameters

All data were chirp corrected prior to kinetic analysis. The fits to data shown in the main manuscript were modelled using an analytical expression comprised from the convolution of a gaussian IRF with multiple exponential rise/decays. The instrument response function was locked to the pump pulse duration determined by PG-FROG.

Table S2 Kinetic fitting parameters for TDPP-Br and TDPP-v-TDPP studied in toluene solution.

Molecule	Probe Wavelength	Rise / fs	τ_1 / fs (Amp/%)	τ_2 / ps (Amp/%)	τ_3 / ns (Amp/%)	R^2
TDPP-Br	615 nm (SE)	IRF limited	103 ± 30 (59)	—	5 ± 1 (41)	0.92
TDPP-Br	750 nm (ESA)	105 ± 5	—	175 ± 5 (73)	5 ± 1 ns (27)	0.99
TDPP-v-TDPP	610 nm (GSB)	IRF limited	—	5.3 ± 0.4 (100)	—	0.98
TDPP-v-TDPP	870 nm (ESA)	92 ± 5	—	5.7 ± 0.4 (100)	—	0.99

4. Electronic Structure Calculations

(a) TDPP

Minimum energy structures for S_0 ($1A_g$), $1B_u$ and $2A_g$ states of simplified TDPP molecule are reproduced from Casal *et al.*.[4]

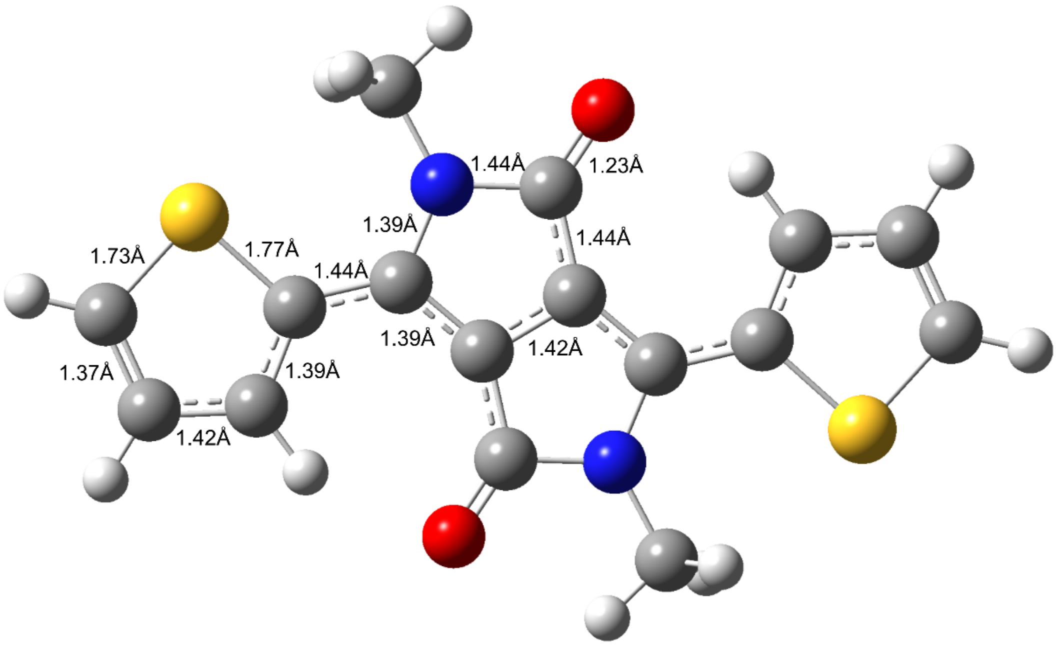

Fig. S6 Minimum energy geometry and associated bond lengths for S_0 ($1A_g$) state of TDPP calculated at the DFT/B3LYP-D3/6-31G(d,p) level of theory.

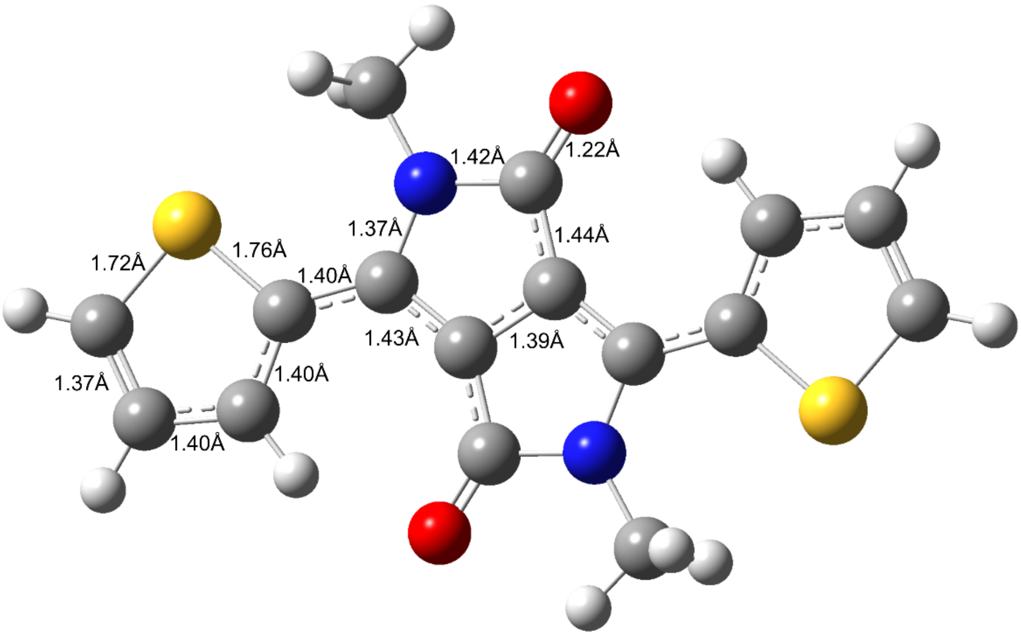

Fig. S7 Minimum energy geometry and associated bond lengths for S_1 ($1B_u$) state of TDPP calculated at the SF-TD-DFT(BHHLYP)/6-31G(d,p) level of theory.

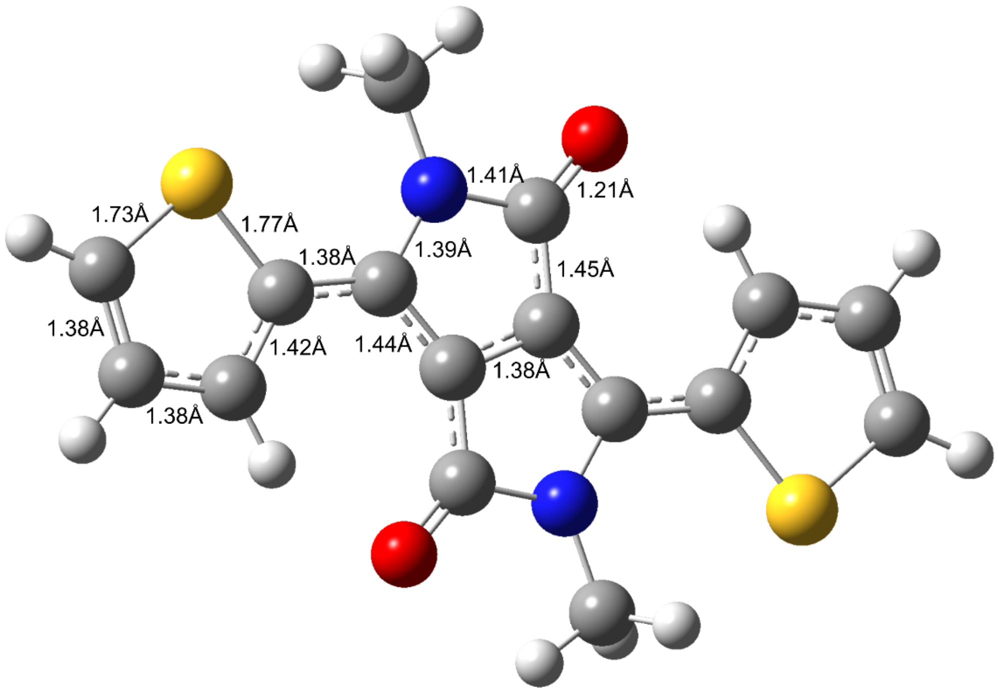

Fig. S8 Minimum energy geometry and associated bond lengths for S_2 ($2A_g$) state of TDPP calculated at the SF-TD-DFT(BHHLYP)/6-31G(d,p) level of theory.

(b) TDPP-v-TDPP

Minimum energy structures for S_0 ($1A_g$), $1B_u$ and $2A_g$ states of simplified TDPP-v-TDPP molecule are reproduced from Casal *et al.*.[4]

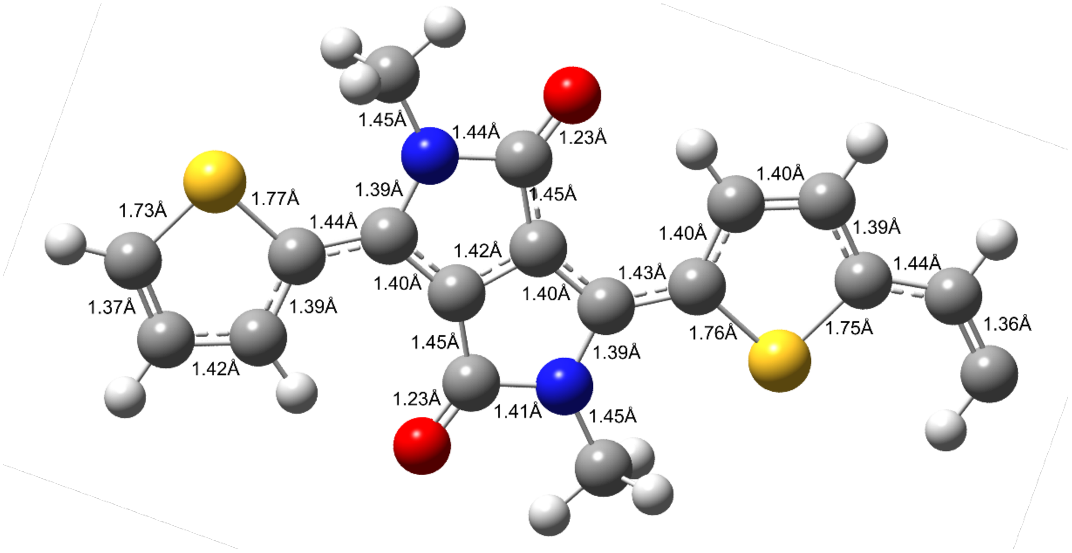

Fig. S9 Minimum energy geometry and associated bond lengths for S_0 ($1A_g$) state of TDPP-v-TDPP calculated at the SF-TD-DFT(BHHLYP)/6-31G(d,p) with enforced C_{2h} symmetry. Only half of the symmetric structure is shown for sake of clarity.

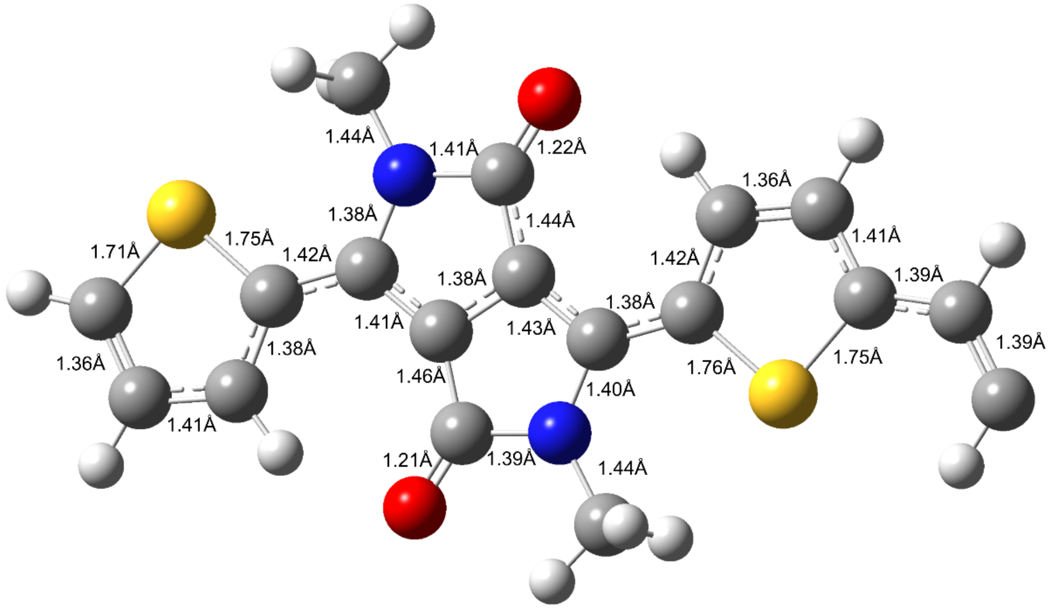

Fig. S10 Minimum energy geometry and associated bond lengths for S_1 ($2A_g$) state of TDPP-v-TDPP calculated at the SF-TD-DFT(BHHLYP)/6-31G(d,p) level of theory with enforced C_{2h} symmetry. Only half of the symmetric structure is shown for sake of clarity.

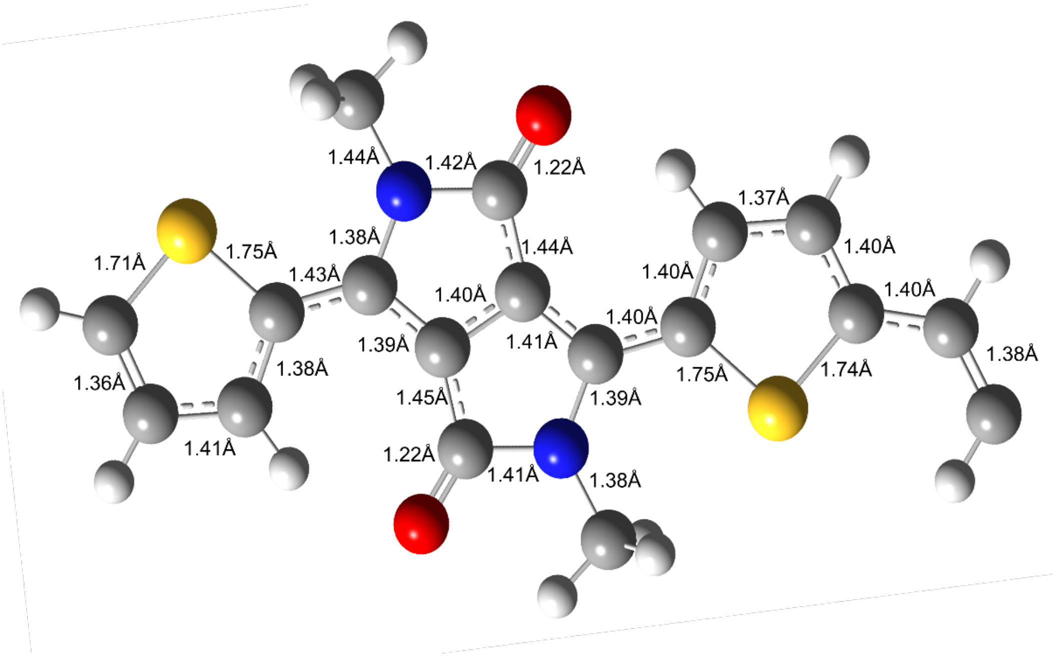

Fig. S11 Minimum energy geometry and associated bond lengths for S_2 ($1B_u$) state of TDPP-v-TDPP calculated at the SF-TD-DFT(BHHLYP)/6-31G(d,p) level of theory with enforced C_{2h} symmetry. Only half of the symmetric structure is shown for sake of clarity.

6. TDPP-v-TDPP and TDPP synthetic methods

Commercially available reagents were purchased and used without further purification unless otherwise stated. 3-(5-Bromothiophen-2-yl)-2,5-bis(2-ethylhexyl)-6-(thiophen-2-yl)-2,5-dihydropyrrolo[3,4-c]pyrrole-1,4-dione was synthesized following the published route.[5] ^{1}H NMR and ^{13}C NMR spectra were recorded on Bruker AV-400 spectrometer in $CDCl_3$ solvent at room temperature. Matrix-assisted laser desorption/ionisation time-of-flight (MALDI-TOF) mass spectrometry was performed on a Bruker ultrafleXtreme MALDI-TOF analyser.

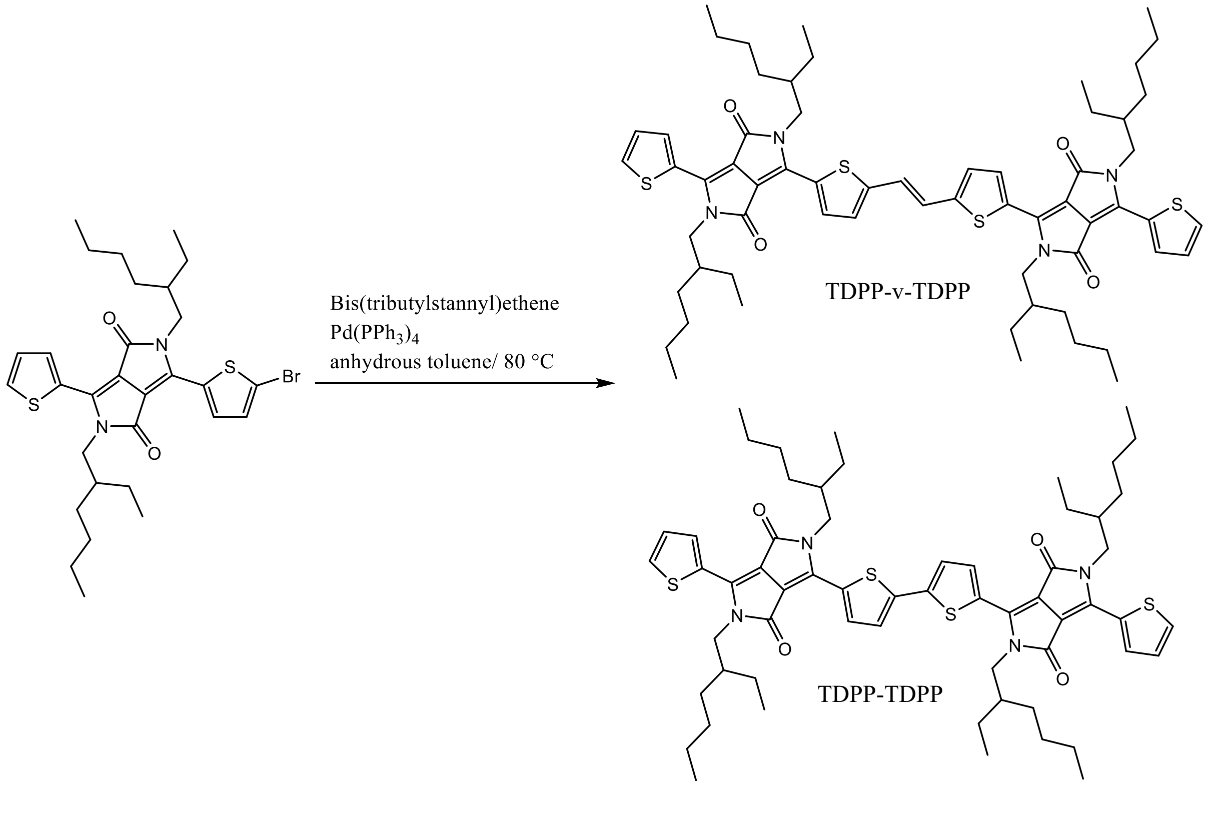

Scheme 1. Synthetic dimerization producing TDPP-v-TDPP and TDPP-TDPP.

Compound 3-(5-bromothiophen-2-yl)-2,5-bis(2-ethylhexyl)-6-(thiophen-2-yl)-2,5-dihydropyrrolo[3,4-c]pyrrole-1,4-dione (0.18 g, 0.30 mmol) and bis(tributylstannyl)ethene (0.08 ml, 0.15 mmol) were dissolved in anhydrous toluene (20 ml) in a 100 ml round bottom flask under nitrogen atmosphere. $Pd(PPh_3)_4$ (0.069 g, 0.060 mmol) was added and the mixture stirred at 80 °C for 16 h. After cooling to room temperature, the organic solvent was removed under vacuum and the crude product was purified by column chromatography over silica (eluent: hexane:dichloromethane (1:1)), followed by preparative recycling GPC using chloroform as the eluent. The product and major by product were isolated as dark purple solids (TDPP-v-TDPP: 0.071 g, yield: 44%; TDPP-TDPP: 0.083 g, yield: 53%).

TDPP-v-TDPP ^{1}H NMR (400 MHz, CD_2Cl_2) δ 8.92 (2H, d, *J* = 4.11 Hz), 8.88 (2H, d, *J* = 4.13 Hz), 7.68 (2H, d, *J* = 5.02 Hz), 7.30 (4H, t, *J* = 4.23 Hz), 7.27 (2H, s), 4.03 (4H, m), 1.87 (2H, m), 1.31 (16H, m), 0.88 (12H, m). ^{13}C NMR (101 MHz, $CDCl_3$) δ 161.79, 161.69, 146.97, 140.31, 139.61, 136.54, 135.57, 130.74, 129.99, 129.44, 128.59, 128.45, 122.86, 108.86, 108.41, 46.10, 39.37, 39.22, 30.46, 30.37, 28.70, 28.50, 23.80, 23.70, 23.23, 23.19, 14.24, 14.13, 10.67, 10.62. MS (m/z): [M+] calcd. for C62H80N4O4S4: 1073.59, Found: 1073.0 (MALDI-TOF).

TDPP-TDPP ^{1}H NMR (400 MHz, CD_2Cl_2) δ 8.92 (2H, d, *J* = 3.89 Hz), 8.87 (2H, d, *J* = 4.16 Hz), 7.71 (2H, d, *J* = 5.07 Hz), 7.47 (2H, d, *J* = 4.19 Hz), 7.30 (2H, t, *J* = 4.49 Hz), 4.01 (4H, m), 1.85 (2H, m), 1.34 (16H, m), 0.88 (12H, m). ^{13}C NMR (101 MHz, $CDCl_3$) δ 161.73, 161.58, 141.11, 138.91, 135.92, 135.26, 133.64, 131.82, 131.11, 129.86, 128.63, 126.94, 109.20, 108.17, 90.53, 46.19, 46.09, 39.28, 39.19, 30.35, 30.28, 28.48, 23.68, 23.17, 14.15, 14.12, 10.61, 10.58. MS (m/z): [M+] calcd. for C60H78N4O4S4: 1047.55, Found: 1070.8 (MALDI-TOF).

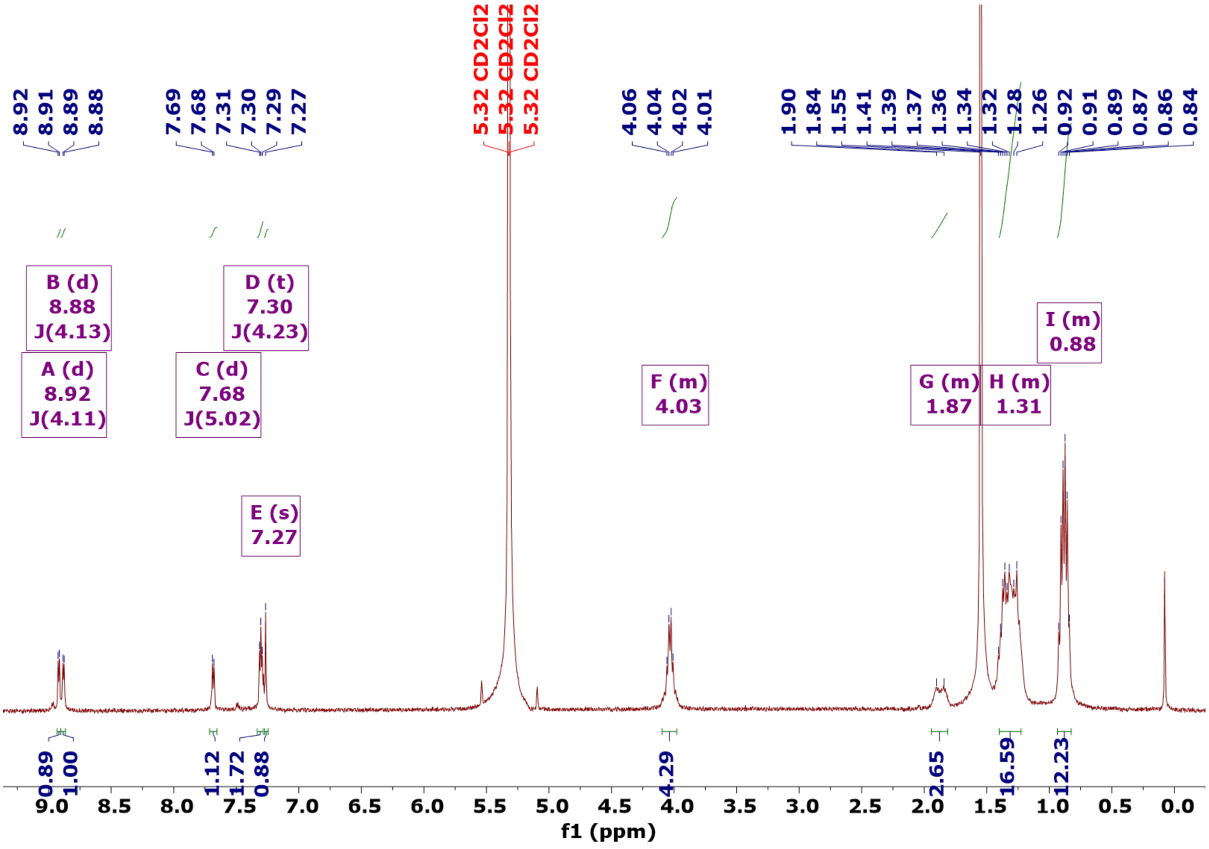

Fig. S12 ^{1}H NMR spectrum of TDPP-v-TDPP

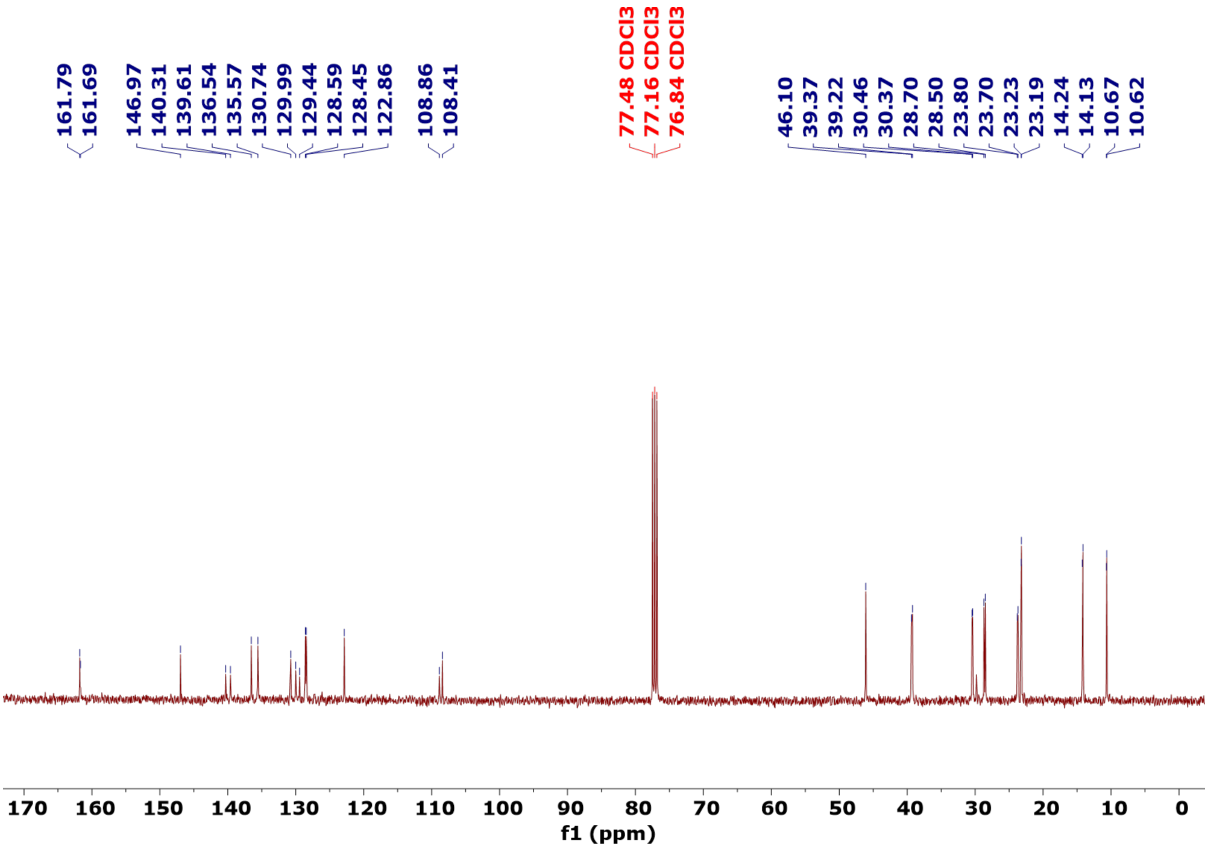

Fig. S13 ^{13}C NMR spectrum of TDPP-v-TDPP

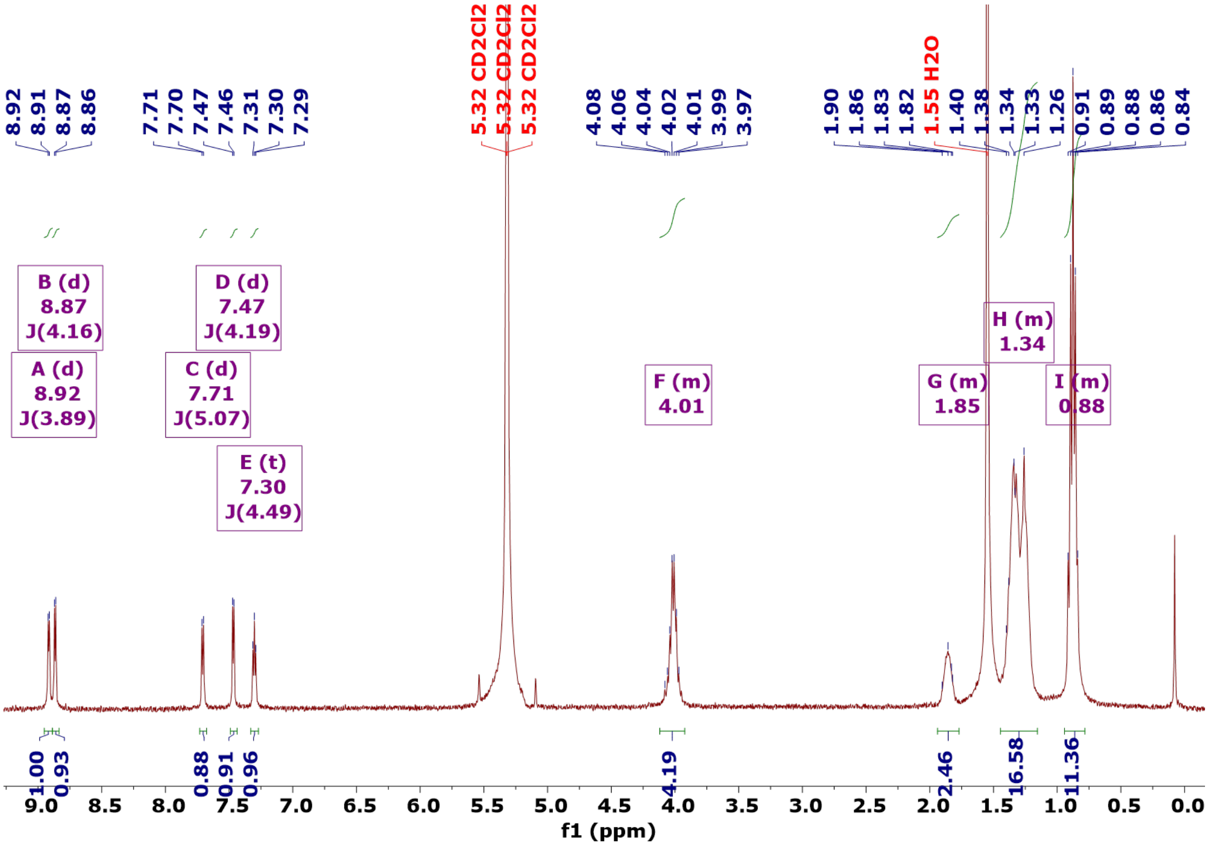

Fig. S14 ^{1}H NMR spectrum of TDPP-TDPP

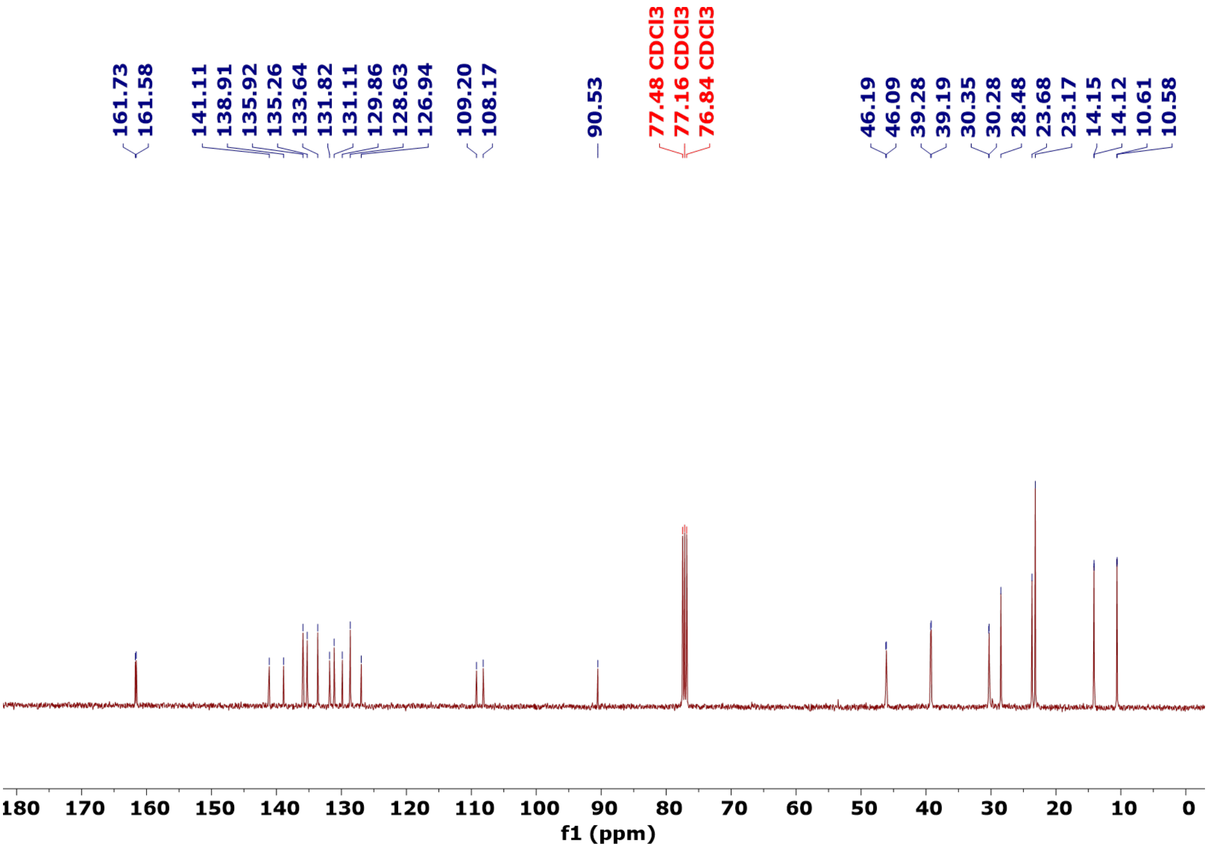

Fig. S15 ^{13}C NMR spectrum of TDPP-TDPP

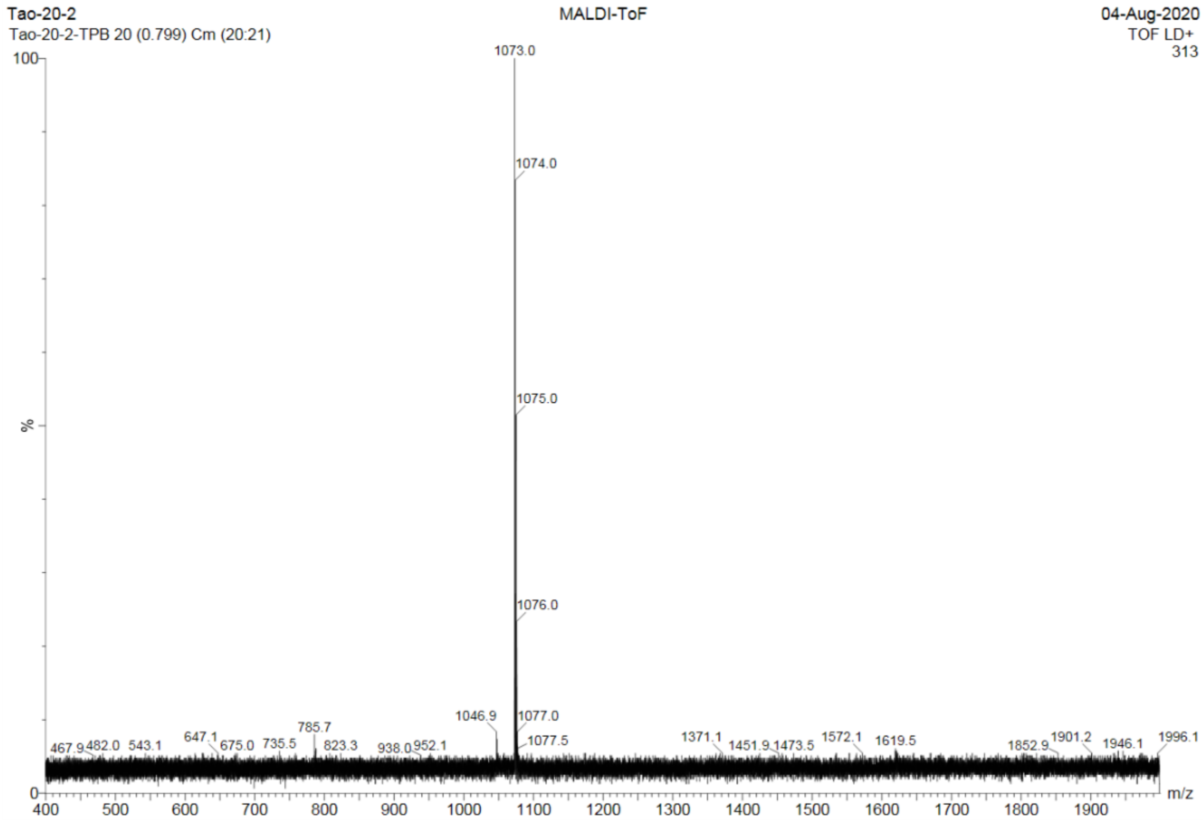

Fig. S16 Mass spectrum of TDPP-v-TDPP

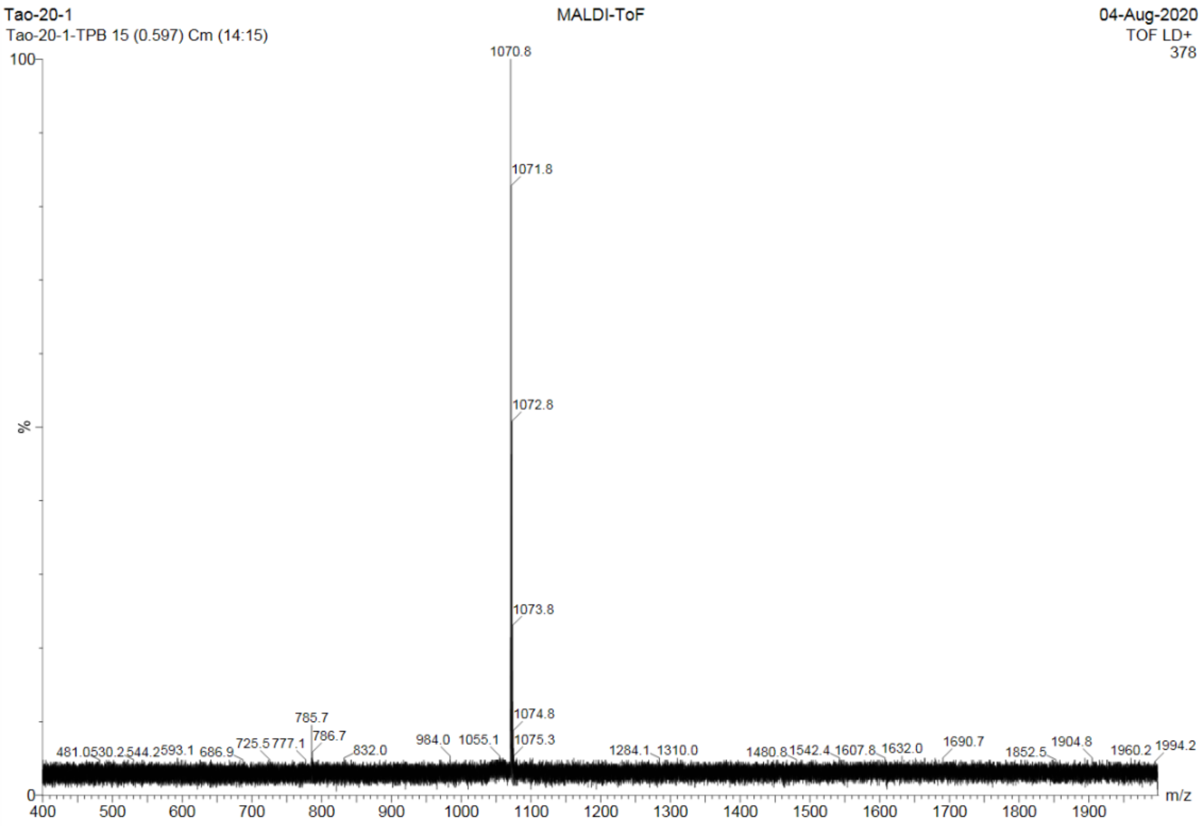

Fig. S17 Mass spectrum of TDPP-TDPP

7. DFT Calculations

To reduce the computational complexity of both DFT/TD-DFT calculations, the effect of truncating the N-alkyl chains on the ground state geometry was investigated. These calculations used the B3LYP functional and a 6-311G(d,p) basis set.

The following substituents were trialled for TDDP-Br: *R* = –H (hydrogen), $–CH_3$ (methyl), $–CH_2CH(CH_3)_2$ (isobutyl), and $–CH_3$, $–CH_2CH(CH_3)_2$ for TDPP-v-TDPP. The calculations were not intended to be exhaustive but to inform whether the geometries associated with short-chain alkyl substituents were representative of the full molecular structure, and to help determine the symmetry point group of molecules. Conformations enforcing planar C_{2h} geometries as well as those without any symmetry constraint were trialled. The latter were found to optimise into structures within the C_i point group (inversion symmetry), where the terminal thiophene moieties rotated out of the molecular plane (as determined by the planar DPP core) by dihedral angle α- see Fig. S18.

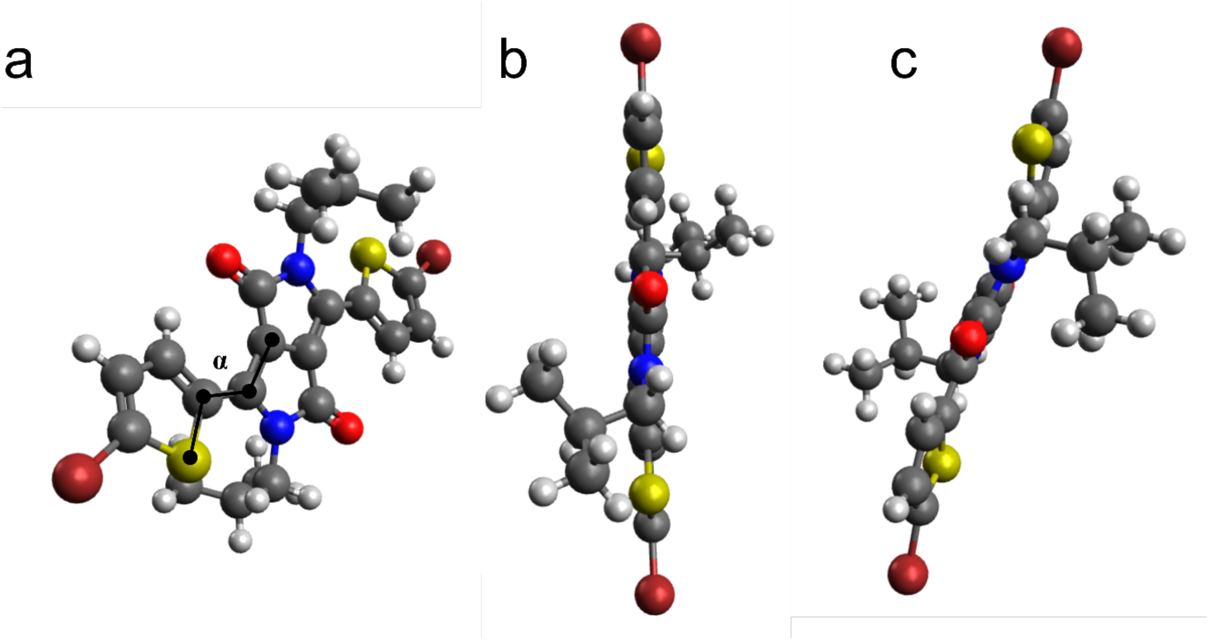

Fig. S18 (a) TDDP-Br structure with illustration of dihedral angle (α), which is defined as the angle the thiophene rings make with the plane defined by the central DPP core. Example structures of α = (b) 0° and (c) 10°.

For the monomer and *R* = –H, calculations predicted the global ground state minimum energy geometry to be planar and C_{2h} (see Table S3). Upon changing the *R* substituent to $–CH_2CH(CH_3)_2$, a sidechain group that is more representative of the true branched alkyl chains

(see Fig. 1 in main paper), an added complication arises from the conformational complexity associated with the isobutyl substituents. In geometries where the hydrogens on the $–CH_2$ group of the isobutyl substituents point towards the thiophene ring (Fig. S19(a,b)), the terminal thiophene rings twist away from the planar DPP core in opposite directions (α = 32°), thus forming a structure which has overall C_i symmetry. While, if the alkyl chains are rotated to avoid steric interactions with the aromatic core of the molecule (Fig. S19(c,d)), α reduces to 6 °. Locking this structure to planarity (C_{2h}) and re-optimisation returns a ground state minimum structure with an associated minimum energy only 11 meV above that of the twisted C_i geometry (α = 6 °). Thermal energy at 298 K is 26 meV, and therefore it is likely that the ground state geometry will quickly interconvert (depending on the barrier height) between these two (and potentially other) minima. Thus, in a time-averaged picture, the planar C_{2h} minima is representative of ground state TDPP-Br.

Calculations for the dimer (full results detailed in Table S3) with methyl and isobutyl *R* groups confirmed similar behaviour to the monomer: the C_{2h} minimum (*R* = –isobutyl) was calculated to be 0.8 meV above that of the global C_i minimum energy geometry- a value which is insignificant compared to solvent fluctuations or thermal energy at 298 K, again justifying approximating a C_{2h} structure for TDPP-v-TDPP.

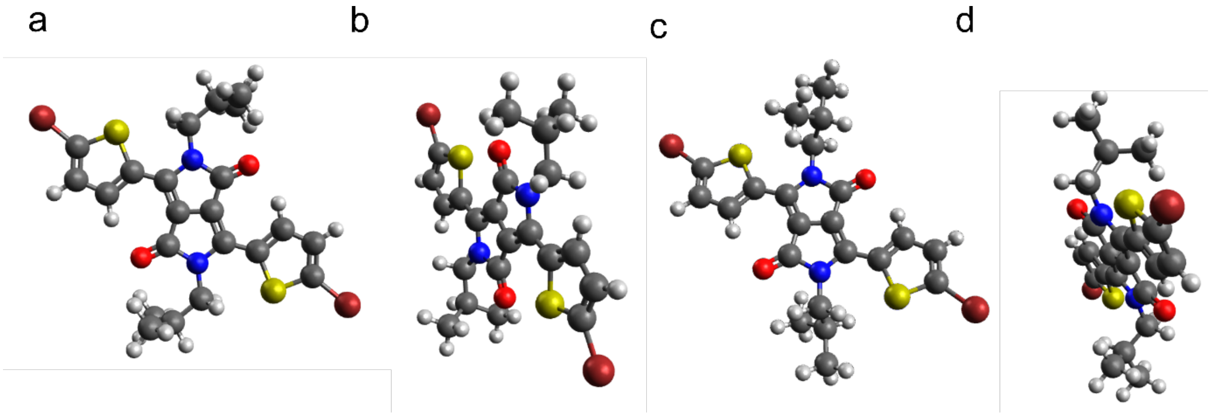

Fig. S19 Two example TDPP-Br geometries (*R* = –isobutyl). (a,b) top-down and side-on perspectives of minimised geometry where the isobutyl side chain CH_2 hydrogens point to the terminal thiophene rings (α = 32°). (c,d) top-down and side-on depictions of the minimum energy where alkyl side chains are rotated to avoid steric interactions (α = 6°).

Table S3. Effect on conformation and side-chain length on TDPP-Br and TDPP-v-TDPP ground state energy and structure. Relative energies are grouped as a function of side chain functionalisation and molecule.

Molecule	Side Chain Functionalisation	Ground state minimum energy / Hartree	Relative Energy / eV	Point Group	α / °
TDPP-Br	Hydrogen	–6736.841270	0.000	C_{2h}	0
TDPP-Br	Methyl	–6815.464659	0.000	C_{2h}	0
TDPP-Br	Isobutyl[1]	–7056.508249	0.755	C_{2h}	0
TDPP-Br	Isobutyl[1]	–7056.521239	0.402	C_i	32
TDPP-Br	Isobutyl[2]	–7056.535996	0.000	C_i	6
TDPP-Br	Isobutyl[2]	–7056.535599	0.011	~C_{2h}	0
TDPP-v-TDPP	Methyl	–3423.266994	0.000	C_{2h}	0
TDPP-v-TDPP	Isobutyl[1]	–3895.102568	0.0566	C_{2h}	0
TDPP-v-TDPP	Isobutyl[1]	–3895.142441	0.0167	C_i	20
TDPP-v-TDPP	Isobutyl[2]	–3895.159159	0.0000	C_i	4
TDPP-v-TDPP	Isobutyl[2]	–3895.158347	0.0008	~C_{2h}	0

[1,2] Alkyl chain conformations shown in Figure S21(a,b), and S21(c,d) respectively.

TD-DFT calculations (again using B3LYP/6-311G(d,p)) returned similar results for the excited bright state ($1B_u$) with isobutyl substituents, where the C_{2h} structure was 14 meV higher in energy than the C_i minima. Calculations for the dimer returned a 30 meV energy difference.

These global minimum energy geometries were used for vibrational normal mode calculations used to assign observed wavenumbers from the wavepacket FT analysis. For these vibrational calculations, R = –CH_3 was used to minimise the computational cost. For TDPP-Br and TDPP-v-TDPP, the vibrational normal modes and associated wavenumbers the S_0 ($1A_g$) and bright excited ($1B_u$) electronic states were calculated using DFT and TD-DFT, respectively. The ground state minimum energy geometries were optimised and vibrational wavenumbers calculated with the DFT exchange-correlation functional B3LYP and a 6-311G(d,p) basis set.

The same basis function and basis set were used with TD-DFT to calculate the associated vibrational wavenumbers of the $1B_u$ excited states.

8. Assignment of vibrational wavepacket wavenumbers

Table S4. Assignment of TDPP-Br vibrational wavepacket modes present in the S_0 trace displayed in Fig. 4.

Wavenumber / cm^{-1}	Associated nuclear motions
161	Symmetric in-plane rocking of the terminal thiophene rings and the DPP core
288	Symmetric in-plane rocking of the terminal thiophene rings relative to the DPP core
372	Symmetric in-plane stretching of both C-Br bonds coupled to a horizontal sheering of the pyrrole rings through the central double bond
505	Symmetric in-plane rocking of the terminal thiophene rings relative to the DPP core
701	Symmetric out-of-plane twisting of the pi-conjugated system with majority amplitude on the DPP core
1071	In-plane asymmetric C-N-C double bond stretching motions
1369	Symmetric C-N-C stretching motions on the central DPP unit coupled to thiophene ring breathing motions
1525	In-plane symmetric C-C double bond over the conjugated backbone with large amplitude on the DPP units

Table S5. Assignment of TDPP-Br vibrational wavepacket modes associated with the $1B_u$ state shown in Fig. 4.

Wavenumber / cm^{-1}	Associated nuclear motions
288	Symmetric in-plane rocking of the terminal thiophene rings and the DPP core
494	Anti-symmetric out-of-plane twisting of the π-conjugated system
705	Symmetric out-of-plane twisting of the π-conjugated system with majority amplitude on the DPP core
1071	In-plane asymmetric C-N-C double bond stretching motions
1362	Symmetric C-N-C stretching motions on the central DPP unit coupled to thiophene ring breathing motions
1416	Symmetric C-C double bond stretching on the central DPP unit coupled to thiophene ring breathing motions
1527	In-plane symmetric C-C double bond over the conjugated backbone with large amplitude on the DPP units

Table S6. Assignment of TDPP-Br vibrational wavepacket modes associated with the $2A_g$ transient shown in Fig. 4.

Wavenumber / cm^{-1}	Associated nuclear motions
965	Anti-symmetric C-S-C stretching mode driven by a large amplitude C-Br stretching motion
1325	Anti-symmetric DPP and thiophene ring breathing modes
1365	Symmetric C-N-C stretching motions on the central DPP unit coupled to thiophene ring breathing motions
1410	Symmetric C-C double bond stretching on the central DPP unit coupled to thiophene ring breathing motions
1479	Anti-symmetric stretching of the central C=C bonds on the DPP unit coupled to C=C stretching modes over the whole conjugated system.

Table S7. Assignment of TDPP-v-TDPP S_0 vibrational wavepacket modes- see Fig. 6.

Wavenumber / cm^{-1}	Associated nuclear motions
215	Symmetric in-plane rocking of the thiophene and the DPP rings
614	Symmetric thiophene ring breathing modes of the terminal thiophene
835	Symmetric thiophene and DPP ring breathing modes across the whole structure
937	Out-of-plane rocking of the hydrogens on the central thiophene rings and vinyl linker
1162	Anti-symmetric stretching of the C=C bonds on the central thiophene rings
1416	Symmetric thiophene and DPP ring breathing modes across the whole structure

Table S8. Assignment of TDPP-v-TDPP 1B_u vibrational wavepackets, data shown in Fig. 6.

Wavenumber / cm^{-1}	Associated nuclear motions
215	Anti-symmetric in-plane rocking of the thiophene and the DPP rings
1467	In-plane C=C double bond stretching modes on the DPP units and terminal thiophene rings.
1502	In-plane C=C double bond stretching modes on the DPP units and terminal thiophene rings.
1531	In-plane C=C double bond stretching modes over the whole pi-conjugated system with larger amplitude on the terminal DPP/thiophene units

Table S9. Assignment of TDPP-v-TDPP 2A_g state vibrational wavepackets- see Fig. 6.

Wavenumber / cm^{-1}	Associated nuclear motions
54	In-plane asymmetric rocking of the DPP units relative to each other through the central thiophenes/vinyl linker.
1107	Low amplitude thiophene and DPP ring breathing motions on all rings in the molecular structure.
1258	High amplitude symmetric C-C double bond stretching modes on the vinyl linker and central thiophene rings.
1422	Symmetric and in-phase thiophene and DPP ring breathing motions on all rings in the molecular structure.
1502	In-plane C=C double bond stretching modes on the DPP units and terminal thiophene rings.

Table S10. Assignment of DPPDTT S_0 vibrational wavepacket modes- see Fig. S5.

Wavenumber / cm^{-1}	Associated nuclear motions
725	In-plane asymmetric C-S-C stretching motion
856	Out-of-plane sheering of the DPP units via a symmetric C=C-C bending motion.
946	In-plane asymmetric stretching motion of the S-C=C-S bonds on the centre unit of the thiophene chain which drives a low amplitude ring breathing motion along the whole thiophene chain.
1072	Thiophene ring breathing mode on the terminal thiophene of the dimer unit

Table S11. Assignment of DPPDTT S_{bright} vibrational wavepacket modes- see Fig. S5.

Wavenumber / cm^{-1}	Associated nuclear motions
727	In-plane asymmetric C-S-C stretching motion coupled to in-plane rocking of the DPP units
1304	DPP and thiophene ring breathing motions throughout the whole molecular structure
1445	DPP and thiophene ring breathing motions throughout the whole molecular structure

Table S12. Assignment of DPPDTT S_{dark} vibrational wavepacket modes- see Fig. S5.

Wavenumber / cm^{-1}	Associated nuclear motions
618	Out-of-plane C=C-C twisting motions on the thiophene chain
703	In-plane asymmetric C-S-C stretching motion coupled to in-plane rocking of the DPP units
734	In-plane asymmetric C-S-C stretching motion coupled to in-plane rocking of the DPP units
859	Low amplitude thiophene and DPP ring breathing modes
946	In-plane asymmetric stretching motion of the S-C=C-S bonds on the centre unit of the thiophene chain which drives a low amplitude ring breathing motion along the whole thiophene chain.

References

1 M. Gustafsson, Spectroscopic Studies of Tissue Using Near-Infrared Raman Microscopy, *Master's Thesis Lund Reports At. Physics, LRAP-207*.

2 J. Kapitán, L. Hecht and P. Bouř, Raman spectral evidence of methyl rotation in liquid toluene, *Phys. Chem. Chem. Phys.*, 2008, **10**, 1003–1008.

3 M. Moser, A. Savva, K. Thorley, B. D. Paulsen, T. C. Hidalgo, D. Ohayon, H. Chen, A. Giovannitti, A. Marks, A. Gasparini, A. Wadsworth, J. Rivnay, S. Inal and I. McCulloch, Polaron delocalization in donor-acceptor polymers and its impact on organic electrochemical transistor performance, *Angew. Chemie*, 2021, **133**, 2–11.

4 M. T. Casal, J. M. Toldo, F. Plasser and M. Barbatti, Using diketopyrrolopyrroles to stabilize double excitation and control internal conversion, *ChemRxiv.* 2022, doi: 10.26434/chemrxiv-2022-2z60p.

5 J. Liu, Y. Sun, P. Moonsin, M. Kuik, C. M. Proctor, J. Lin, B. B. Hsu, V. Promarak, A. J. Heeger and T. Q. Nguyen, Tri-diketopyrrolopyrrole molecular donor materials for high-performance solution-processed bulk heterojunction solar cells, *Adv. Mater.*, 2013, **25**, 5898–5903.
